# A randomised, placebo-controlled trial in healthy humans of modified cellulose or psyllium evaluating the role of gelation in altering colonic gas production during inulin co-administration

**DOI:** 10.1039/d5fo03532e

**Published:** 2026-02-04

**Authors:** Joshua E. S. J. Reid, Alaa T. Alhasani, Thomas MacCalman, Daniel Amor, Abdulsalam I. Aliyu, Amisha A. Modasia, Hannah Harris, Frederick J. Warren, Caroline Hoad, Penny A. Gowland, Gleb E. Yakubov, Colin Crooks, Maura Corsetti, Luca Marciani, Robin C. Spiller

**Affiliations:** a Food Materials Research Group, School of Bioscience, University of Nottingham Sutton Bonington UK joshua.reid@nottingham.ac.uk; b Nottingham NIHR Biomedical Research Centre and Nottingham Digestive Disease Centre, School of Medicine, University of Nottingham Nottingham UK robin.spiller@nottingham.ac.uk; c Faculty of Health and Rehabilitation Sciences, Princess Nourah Bint Abdul Rahman University Riyadh Saudi Arabia; d Quadram Institute Bioscience, Norwich Research Park Norwich UK; e Sir Peter Mansfield Imaging Centre, School of Physics and Astronomy, University of Nottingham Nottingham UK; f Food Biopolymers Laboratory, School of Food Science and Nutrition, University of Leeds Leeds UK

## Abstract

Dietary fibre is vital for a healthy diet, yet many people avoid it because of symptoms induced by colonic gas. Slowing rapid fermentation decreases colonic distention and reduces symptoms, allowing for better tolerance of prebiotics. Co-administration of inulin, a fermentable fibre, with psyllium, a gel-forming fibre, reduces gas production in irritable bowel syndrome patients compared to administering inulin alone, but the underlying mechanism is unclear. We hypothesise that psyllium polysaccharides’ physically cross-linked gel resists gastrointestinal shear forces and impairs microbial access to inulin, thereby delaying fermentation. Methylcellulose is another physically cross-linked fibre ingredient, widely used in food production for its tunability and affordability. Our aim was to develop a preparation of methylcellulose of comparable functionality to psyllium. A formulation of methylcellulose with comparable rheological and inulin release behaviour was developed *in vitro*. We subsequently performed a randomised, three-way, placebo-controlled non-inferiority study with healthy volunteers (*n* = 30), comparing the slowing of fermentation of inulin by co-administering with psyllium, methylcellulose or a control maltodextrin. Fermentation *in vivo* was assessed by breath hydrogen measurements for 24 hours after ingestion. While psyllium significantly reduced initial breath hydrogen production compared to the placebo, a non-inferior effect on reduction in initial breath hydrogen with methylcellulose was not demonstrated. Despite similar physicochemical properties, psyllium and methylcellulose hydrogels exhibited different transit behaviour based on the breath hydrogen time to rise >10 ppm and time to peak. We hypothesise that the fast reformation of psyllium's polysaccharide network or “self-healing” properties after deformation by intestinal pressure waves may underpin its effectiveness in slowing fermentation. The clinical trial registry number is NCT05911347 (https://clinicaltrials.gov).

## Abstract 2

Dietary fibre is vital for a healthy diet, yet many people avoid it because of symptoms induced by colonic gas. We explore how gel-forming, poorly fermented fibres affect the colonic gas produced by ingesting rapidly fermentable fibre.

## Graphical abstract

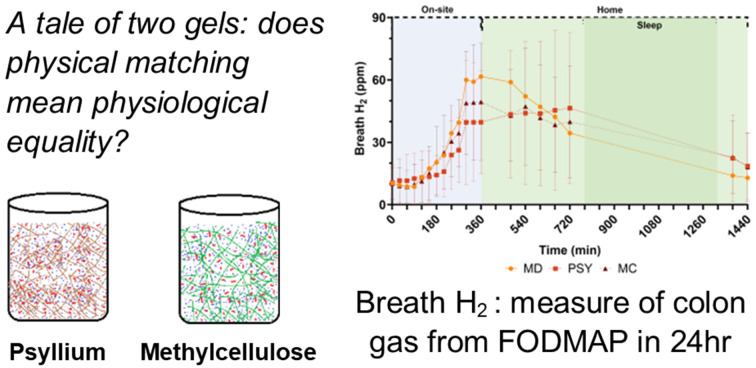

## Introduction

Despite the clear epidemiological and clinical trial evidence that dietary fibre intake improves health outcomes including obesity, type II diabetes, cardiovascular disease and colon cancer,^1,2^ fibre intake in Europe and the USA remains approximately half the recommended amount of 25 g per day.^3^ There are many reasons why consumers do not achieve this target, including the lack of awareness of positive benefits and socio-economic barriers.^4–6^ Recent changes in consumer preferences towards low-carbohydrate diets may also be a significant factor^4^ since these diets may be beneficial in addressing other increasingly important health concerns such as obesity or type II diabetes.^7^ Another recent factor is the promotion of low FODMAP (fermentable oligo-di–mono-saccharide and polyhydric alcohol) diets for the treatment of irritable bowel syndrome (IBS).^8,9^ While this reduces symptoms, the restrictive nature of the diet, unless supervised by a dietitian, leads to reduced fibre intake.

Our aim is to combine different types of fibres to retain the metabolic benefits whilst minimizing the undesirable gas associated symptoms. Magnetic Resonance Imaging (MRI) studies have demonstrated that the smaller molecular weight carbohydrates, like fructose, increase small bowel water^10^ while high molecular weight FODMAPs like inulin predominately affect the colon, increasing colonic volume and gas content which correlate with the symptoms of bloating and gas.^11^ Adding psyllium to inulin delays orocaecal transit and reduces the rise in breath hydrogen and colonic gas volumes in IBS patients;^12^ however its mode of action remains unclear.

Psyllium (PSY) is made up of highly branched non-starch polysaccharides and forms a viscoelastic physical gel when added to water. When taken in doses ranging from 3.5 to 21 g daily, it impedes water absorption, increasing small bowel water content 2–3 fold.^13^ This material enters the colon, increasing colon volumes and ascending colon T1, a MRI measure of colonic water,^14^ which leads to a softening of stool and relief of symptoms of constipation.^15^ From recent *in vitro* fermentation models of PSY fibre and its various fractions in solution it has been shown to be slowly fermented by colonic bacteria so that only small amounts remain in stool.^12,16^

We have recently shown that the delay in colonic fermentation of inulin due to PSY could be mimicked by dividing inulin into multiple doses over 6 hours. However, in the subsequent 24 hours the divided dose actually caused greater gas production than PSY treatment.^17^ This suggested that the reduction in gas production is not solely due to slowing delivery to the ascending colon but also because PSY acts in both the colon and the small bowel. Building on these findings we hypothesised that it acts by forming a gel, trapping inulin and delaying bacterial access, thereby slowing but not preventing colonic fermentation. At low levels of production, most hydrogen diffuses through the colon mucosa and is absorbed without causing symptoms. Around 65% is excreted in breath and 35% passed as flatus; however if rates of production exceed 200 ml in a 24 hour period the colon becomes distended and the amount excreted as flatus rises to 75%.^18^ Thus reducing the maximum rate of fermentation, without altering total fermentation during passage through the colon, might well reduce symptoms and still expose the entire colonic mucosa to the beneficial effects of microbial metabolites such as short chain fatty acids (SCFAs), especially butyrate.^19^ This could retain the metabolic benefits associated with SCFAs (reducing obesity/improving metabolic syndrome) while reducing adverse effects associated with excessive gas.

To assess our hypothesis, we compared the performance of PSY against a model gel-forming dietary fibre preparation, which could form a reversible cross-linked gel network that would act to alter the colonic fermentation of a model FODMAP. Cellulose derivatives are widely used in the food industry to control the viscoelasticity flow and gel behaviour of a wide range of food products. They can also be produced at a much greater scale than psyllium which is derived from *Plantago ovata*, predominantly cultivated in western India with reported yields of 720 kg ha^−1^.^20^ In contrast, celluloses derived from wood pulp can be produced in greater quantities as part of forestry biorefinery designs, such as with Nordic poplar at 2400 kg ha^−1^.^21^ One cellulose derivative, methylcellulose (MC), can undergo a reversible sol–gel transition when heated to produce a hydrogel network driven by preferential interaction of the hydrophobic methoxy functional groups on MC.^22^ Furthermore, it has been shown to be poorly fermented using *in vitro* models of the human colonic microbiota.^23^

Our aim was to mimic the gel-forming and viscoelastic properties of PSY using a preparation of MC, to test our hypothesis that gelation accounts for the slowing of colonic fermentation of a model FODMAP. The characterisation of the hydrogel properties of different preparations of MC was compared with that of PSY to define a formulation for our randomised, placebo-controlled trial. Our objective was to determine whether MC was non-inferior to PSY in reducing the production of breath hydrogen (BH_2_) *versus* placebo (maltodextrin, MD) in a 6-hour period post-ingestion. From the changes in BH_2_ post-ingestion, we can also infer transit effects of PSY and MC against the placebo based on time required for a sustained rise and time required to reach peak BH_2_.^11,17^ Our previous work had suggested that those with a short whole gut transit time (WGTT) show a lesser effect of psyllium, so we also assessed WGTT using the blue muffin test as previously described.^17^ This previous study showed that monitoring BH_2_ over a 24-hour period is sufficient to allow a return to the baseline, allowing us to assess whether MC or PSY results in a delayed but not a loss of colonic fermentation after ingestion of inulin as compared to MD.

## Materials and methods

### Materials

Inulin (Orafti® HP, Beneo, Mannheim, Germany), psyllium husks (Buy Wholefoods Online, Ramsgate, United Kingdom), maltodextrin (GLUCIDEX® 2, Roquette UK Ltd, London, United Kingdom) and methylcellulose (Benecel® A4M (4000 cP) and MX (40 000 cP), IMCD UK Ltd, Sutton, United Kingdom) were obtained for physicochemical characterisation and dietary intervention studies. Steviol glycoside (Stevia Sweet, Stevia Shop, Castleford, UK) and strawberry glycidate (CAS 77-83-8, Sensory Lab, London, UK) were obtained for the dietary intervention studies. Vanillin (CAS 121-33-5, 99%) and sulfuric acid (CAS 7664-93-9, 95%) were obtained from Merck Life Science UK Limited for inulin quantification for the *in vitro* experiment.

Details on the preparation and physicochemical characterisation of dietary fibre solutions are provided in the SI.

### Intervention study

#### Study overview and endpoints

This research was approved by the Research Ethics Committee in the Faculty of Medicine and Health Sciences (FMHS) at the University of Nottingham on July 2022 (Ethics Reference No. FMHS 19-0622) and was conducted in compliance with Good Clinical Practice (GCP) guidelines from the National Institute for Health Research (NIHR) and the Declaration of Helsinki. This study was registered at https://www.ClinicalTrials.gov (ID: NCT05911347). Details on participant recruitment and randomisation are provided in the SI.

The intervention study was a single centre, single-blinded, randomised three-arm crossover trial conducted from April to August 2023 at the Nottingham Clinical Research Facility (N-CRF), Queens Medical Centre, Nottingham, UK. The study compared the effects of two gel-forming preparations, psyllium husk (PSY, 15 g) and methylcellulose (MC, 15 g), and a non-gelling carbohydrate placebo maltodextrin (MD, 15 g) on the colonic fermentation of inulin (IN, 15 g), as inferred from changes in measured breath hydrogen and methane over a 24-hour period. All interventions were prepared in 375 g water. To improve the acceptability of the dietary intervention we added stevia at a low concentration (at 187.5 mg per serving). The effects of steviol glycosides on digestion physiology and the gut microbiome have been discussed previously;^24,25^ however a recent dietary intervention study showed that daily consumption of stevia-sweetened beverages for four weeks did not lead to a significant change in either the gut microbiome composition or short chain fatty acids present in fecal samples.^26^ In comparison with the 15 g dose of inulin in each of the dietary interventions, we anticipate the effect of stevia on the gut microbiome to be minor.

The primary endpoint of the study was to assess whether MC is non-inferior to PSY in reducing the BH_2_ area under curve in the first six hours post-ingestion (AUC_0–6 h_) compared to the placebo MD.

The secondary objectives of the study were:

A. To assess BH_2_ production over the total (0–24 h) post-ingestion period.

B. To measure orocaecal transit time (OCTT, BH_2_ analysis) and whole transit time (WGTT, blue muffin test).

C. To assess breath methane (BCH_4_) production over the initial (0–6 h) and total (0–24 h) post-ingestion period.

#### Study day overview

Participants were contacted by e-mail 24 hours prior to attending a study day to remind them to adhere to the dietary and lifestyle restrictions (for details, see the SI). Upon arriving nil by mouth, the participants were instructed to brush teeth and rinse their mouth with an alcohol-free mouthwash.

Breath analysis for hydrogen (BH_2_) and methane (BCH_4_) was performed using a Gastrogenius breath analyser (Laborie, Portsmouth NH, USA). Measurement was taken at the baseline, where a BH_2_ concentration of <30 ppm was required to begin the study day; if concentrations were greater than 30 ppm, the participants were offered one further opportunity after cleaning their mouth a second time; however if after a second time the BH_2_ was greater than 30 ppm, the participants were sent home and their study day rescheduled. The participants also completed a modified Gastrointestinal Symptoms Rating Scale (GSRS) as previously described.^11^

After baseline measurements, the participants consumed their allocated intervention meal. The intervention meal was portioned into three servings and provided with 100 mL water per serving (total 300 mL). The participants had 25 minutes to consume all three servings. Details on intervention preparation is provided in the SI. A standard meal (tomato & mozzarella pasta meal, 500 kcal) and 300 mL water were provided to the participants 210 minutes after consuming the intervention meal. The fructan content of the meal was low compared to that in the 15 g inulin challenge (approximately 2 g per serving^27,28^) and was consistent across each arm of the study.

**Fig. 1:**
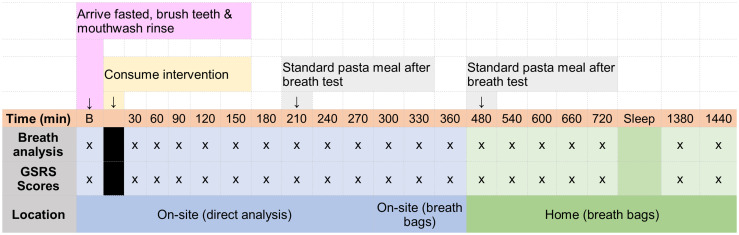
Study day schedule showing the frequency of breath analysis and symptom reporting across the study site and at home, B = baseline.

After consuming the intervention meal (time 0), measurements of BH_2_ and BCH_4_, as well as GSRS scores, were taken at 30-minute intervals over a 6-hour period on site using direct breath sampling into the Gastrogenius breath analyser. In addition, the participants were provided with 8 airtight breath bags to collect breath samples at home (5 samples in the evening of the study day and two samples the morning after and one spare bag). The breath bags were then returned to the study centre and processed using the Gastrogenius breath analyser. A summary of each study day has been provided in Fig. 1.

Orocaecal transit time (OCTT) was calculated from the time for BH_2_ to show a sustained (>60 min) rise of >10 ppm.^17^ Additionally, the time to peak H_2_ (TTP BH_2_) measured was also used to assess the combined effects of alteration in both time to reach the colon and metabolism of fermentable carbohydrates by the colonic microbiota.^11^

WGTT was assessed on a separate day prior to any intervention by subjects ingesting a muffin with blue food colouring and recording when their stool colour changed as previously described.^17,29^

## Data analysis and statistics

All statistical tests were performed using GraphPad Prism V10. Distribution of data was assessed for normal distribution; normally distributed data were reported as mean ± SD while non-normally distributed data were reported as the median and interquartile range (IQR). For comparison across groups where data were not normally distributed, a transformation of the data using a Box–Cox model was applied to normalise the distribution allowing the use of the ANOVA statistical test.^30^ The effect of time and fibre treatment together on measured BH_2_ was assessed by a repeated measures two-way ANOVA adjusting for the interaction between fibres and time, and within patient correlation. For all primary and secondary outcomes, repeated measures one-way ANOVA tests of within-patient differences were applied for comparisons of each treatment (*i.e.* PSY *vs.* MC *vs.* MD), whereas Student's paired *t*-tests were used for comparisons of treatment effects (*i.e.* differences from the placebo). Period effects were also tested for using the two sample *t* test of the semi-period differences. Log rank tests were used to compare transit times as not all patients achieved a complete transit in the study period and were therefore censored at the study end.

### Sample size estimation

The primary objective of the study was to determine whether MC is non-inferior to PSY in reducing the BH_2_ area under the curve in the first six hours post-ingestion (AUC_0–6 h_). Given the more favourable characteristics of MC, we would accept a lesser treatment effect and we chose based on earlier work a non-inferiority margin of 3116 ppm min^−1^ for the reduction in breath hydrogen.^17^ Therefore, the MC intervention would be judged to be non-inferior to PSY if the lower bound of the 95% CI of the difference of methylcellulose from the placebo did not exceed 3116 ppm min^−1^ less than the mean difference of psyllium from the placebo. To detect this with 80% power and 0.05 alpha we required 30 subjects.

## Results

### Physicochemical characterisation of dietary fibre gels

#### *In vitro* model of inulin release from hydrogels

**Fig. 2:**
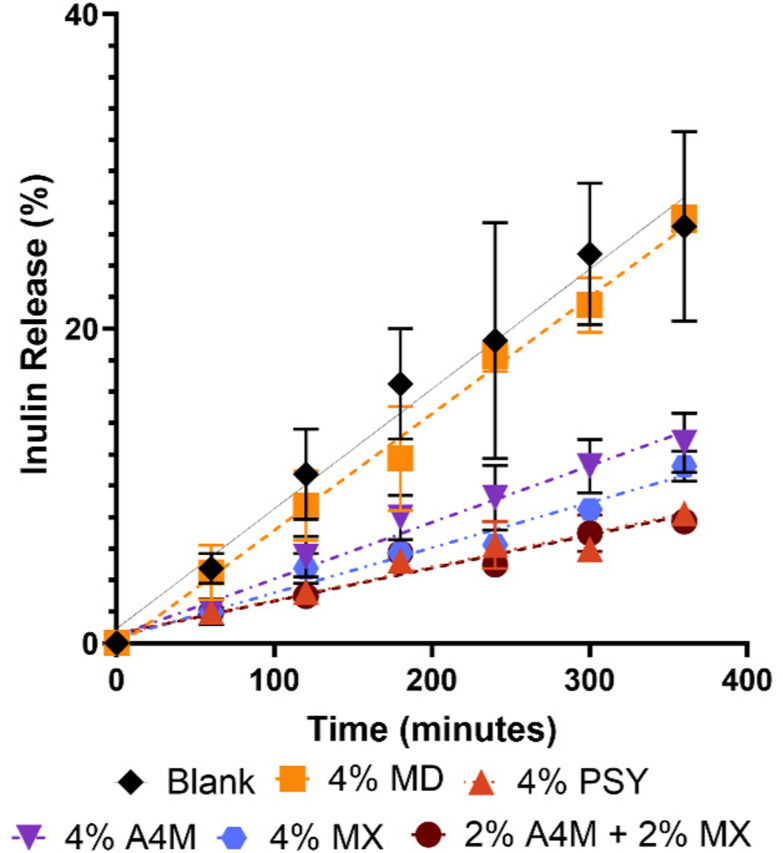
Effect of different carbohydrate substrates on the release of inulin using an *in vitro* dialysis model. Inulin concentration in dialysis buffer was determined using a modified vanillin colorimetric method.^31^ All points represent the mean of triplicate measurements. Data table from the figure provided in the SI.

Over a 6-hour period, the quantity of IN released was successfully measured, with the change in concentration largely following a linear, first order rate of release for all samples (Fig. 2). As expected, the blank sample showed the highest amount of IN released; however the percentage of IN released after 6 hours was only 28% of the original inulin placed into dialysis tubing. The maltodextrin sample showed a comparable amount of inulin released, with 27% of the initial IN content being measured in the dialysis buffer after 6 hours. The psyllium sample showed a distinctly lower concentration of released inulin, with only 8% of the original inulin amount detected after 6 hours.

Three different preparations of MC were assessed to compare the efficacy on release of IN: (1) 4% A4M, (2) 4% MX, ands (3) 2% A4M + 2% MX. The A4M-only and the MX-only preparations showed 14% and 12% of original inulin released after 6 hours, respectively. Interestingly, the MC preparation using both A4M and MX grades showed the lowest concentration of released inulin after 6 hours, with an equivalent of 8% of original inulin amount detected.

#### Flow properties of dietary intervention hydrogels

**Fig. 3:**
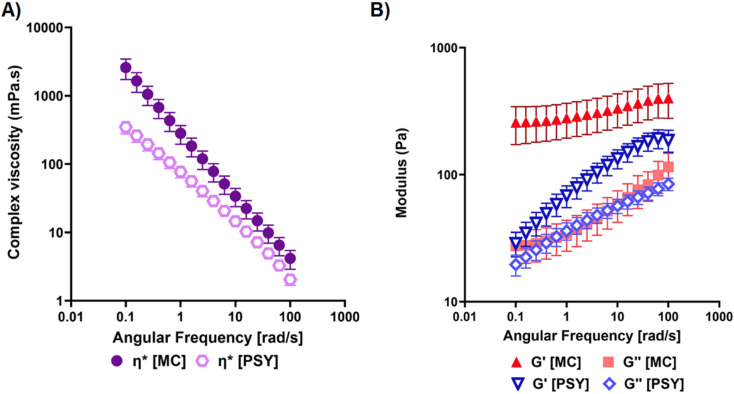
Characterisation of the flow behaviour of candidate dietary fibre hydrogels under frequency sweep tests at 37 °C: (a) apparent complex viscosity, (b) storage (*G*′) and loss (*G*″) moduli.

The relative viscoelastic flow behaviours of the PSY and MC hydrogels are reported in Fig. 3. Due to the heterogeneous nature of PSY hydrogels, a cup and vane configuration was used for characterising its flow properties. In contrast, MC forms a homogeneous hydrogel which, on consumption, will be broken up through oral processing. To standardise the effect of oral processing on the *in vivo* flow behaviour of the MC preparations, a domestic food blender was used to fracture the homogeneous hydrogel, forming gel particles which were less than 10 mm in diameter.

The apparent complex viscosity, η*, was measured for both hydrogel materials with biological replicate measurements performed across a frequency range of 0.1–100 rad s^−1^ (Fig. 3A). Both showed a decrease in η* with increasing test frequency, with a greater η* observed for the MC hydrogels across the measured frequency range. The contributions to η*, namely the storage modulus, *G*′, and the loss modulus, *G*″, demonstrate a more distinct difference between the two hydrogels (Fig. 3B). While *G*″ (the viscous contributions to viscoelastic behaviour) is similar in these hydrogels in this test, the magnitude and trend of *G*′ (the elastic contributions to viscoelastic behaviour) are noticeably different (Fig. 3). For PSY, we see an increase in *G*′ with increasing test frequency, whereas MC shows little variance in *G*′, as well as exhibiting greater *G*′ values than PSY over the measured frequency range.

### Intervention study results

In total, 41 volunteers were randomised to the study. Thirty completed all three study arms and were included in the data analysis (see the Consort diagram in the SI). All participants were healthy adults with only one smoker. The median age was 27 years (IQR: 24–32), with 57% female participants.

### Primary endpoint – non-inferiority of methylcellulose compared to psyllium

Non-inferiority was assessed by comparing the treatment effect *i.e.* the difference of the BH_2_ AUC_0–6 h_ for each intervention compared to the placebo (*i.e.* PSY – MD *vs.* MC – MD). The treatment effect of MC was found to be 1136 ppm with a 95% CI of (−650, 2921), while for PSY it was 2506 ppm with a 95% CI of (450, 4563). The difference between the lower confidence interval for MC and the mean of PSY was 3156, which just exceeded our pre-defined non-inferiority margin of 3116 ppm (ref. 32 and 33); thus the non-inferiority of MC compared to that of PSY was not demonstrated.

### Secondary endpoints

#### Breath hydrogen production

**Fig. 4:**
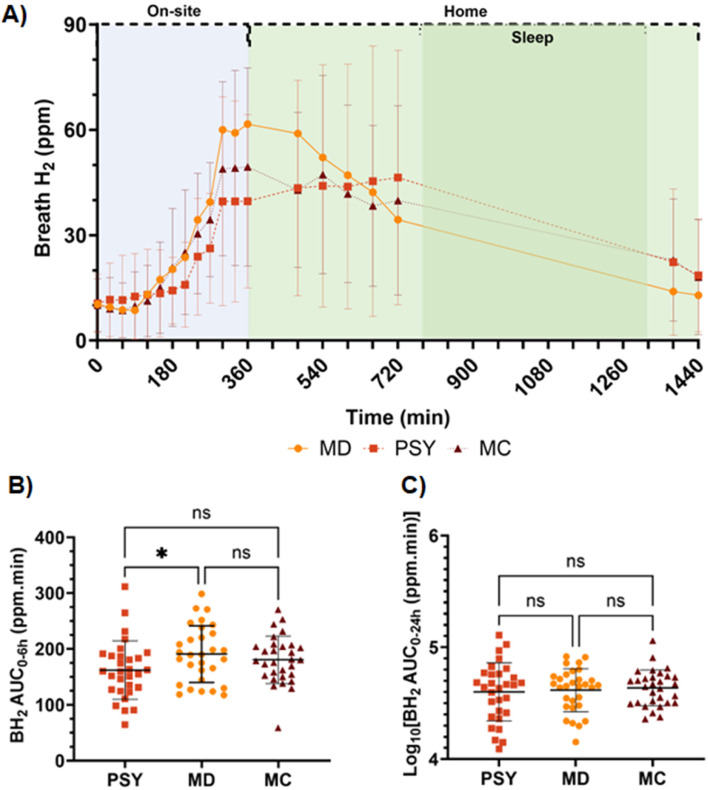
Breath hydrogen (BH_2_) data (*n* = 30) for the three study arms; maltodextrin placebo (MD), psyllium husk (PSY) and methylcellulose (MC). (A) time curve across a 24 h study period. Data shown as mean and standard deviation with the location and method of sampling (on-site = direct, home = breath bag). (B) Transform (Box–Cox, *λ* = 0.5) of BH_2_ AUC_0–6 h_ (ANOVA, *p* = 0.020, Tukey PSY *vs.* MD, *p* = 0.033). (C) Transform (Log_10_) of BH_2_ AUC_0–24 h_ (ANOVA, *p* = 0.63). Data transformed to normalise distribution prior to statistical analysis.

Production of BH_2_ after the intervention challenge was monitored over 24 hours (Fig. 4A). From the two-way ANOVA test, a significant interaction between the fibre treatment and time was observed on the measured BH_2_ (*p* < 0.0234). The BH_2_ AUC_0–6_ and AUC_0–24 h_ were not normally distributed; therefore, a Box–Cox transformation (*λ* = 0.5) was applied to allow application of one-way repeated measures ANOVA test. There was a significant reduction in the transformed BH_2_ AUC_0–6 h_ for PSY *versus* MD (*p* = 0.033) but not for MC *versus* MD (*p* = 0.49) or for PSY *versus* MC (*p* = 0.17, *post-hoc* Tukey's multiple comparisons test) (Fig. 4B). However, there was no significant difference in the transformed BH_2_ AUC_0–24 h_ for PSY compared to that for MD (*p* = 0.90), for MC compared to that for MD (*p* = 0.84) or for PSY compared to that for MC (*p* = 0.69) (Fig. 4C).

#### Gastrointestinal transit

Whole gut transit time (WGTT) was assessed by the blue muffin test prior to any treatment and gave a median 22.8 hours (IQR 18.9–40.1). Of the 30 participants, 4 demonstrated fast WGTT (less than 14 h), 4 demonstrated slow WGTT (more than 59 h) and 22 demonstrated normal WGTT (14–59 h).^29^

##### Oro-caecal transit (OCTT)

**Fig. 5:**
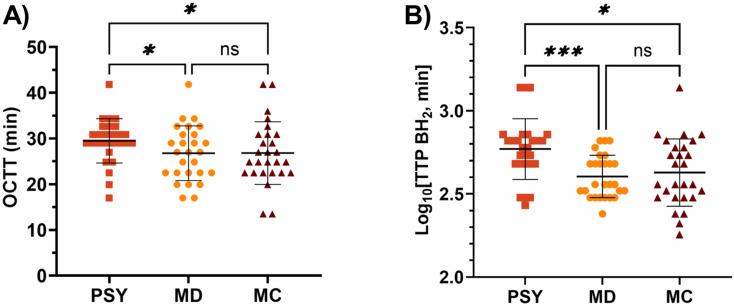
Gastrointestinal transit inferred from changes in BH_2_ data. (A) Transform (Box–Cox, *λ* = 0.5) of (OCTT) in minutes, ANOVA; *p* = 0.013, Tukey; PSY *vs.* MD *p* = 0.046, PSY *vs.* MC *p* = 0.033. (B) Transform (Log_10_) of TTP BH_2_ in minutes, ANOVA; *p* = 0.001, Tukey; PSY *vs.* MD *p* < 0.001, PSY *vs.* MC *p* = 0.029. Data transformed to normalise distribution prior to statistical analysis.

Of the 30 participants, 27 were included in statistical analysis; 2 participants were excluded due to a non-significant rise in BH_2_ over 24 h, and 1 participant was excluded due to baseline breath H_2_ exceeding the pre-defined limit of 30 ppm. OCTT increased significantly with PSY compared to both MD control and MC (Fig. 5A). Furthermore, there was a highly significant increase in normalised Time to Peak (TTP) BH_2_ for PSY compared to those of both MD (*p* = 0.0006) and MC (*p* = 0.029), whereas MD and MC were not significantly different (*p* = 0.75) (Fig. 5B).

### Exploratory endpoints: correlation between transit and breath hydrogen

**Fig. 6:**
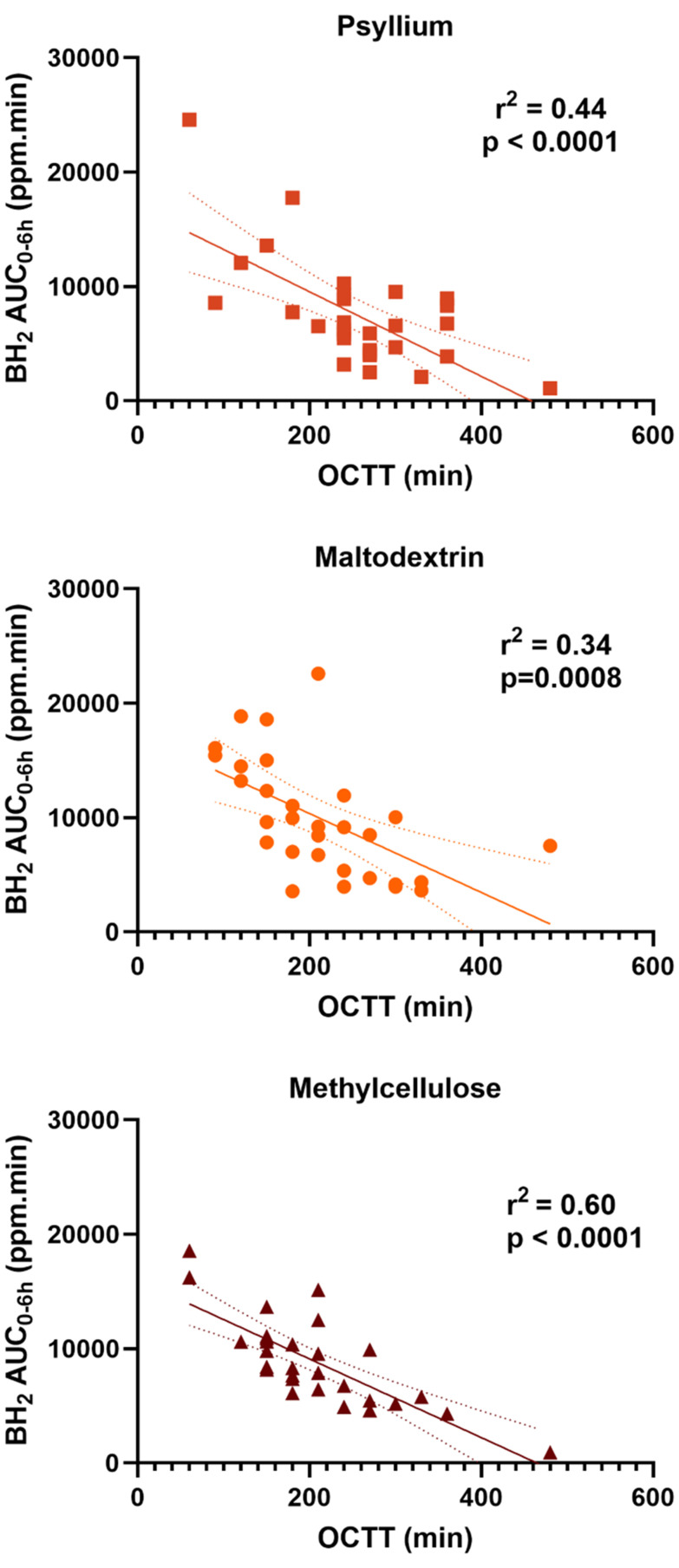
Correlation between BH_2_ AUC_0–6 h_ and OCTT for PSY, MD and MC.

There were strong negative correlations between OCTT and BH_2_ AUC_0–6 h_ for all 3 interventions accounting for 44% of the variance in BH2 AUC_0–6 h_ for PSY, 34% for MD and 60% for MC (Fig. 6). There was however no significant correlation between OCTT and AUC_0–24 h_ for either of the interventions (data not shown). There was also no significant correlation between WGTT and AUC_0–6 h_ nor AUC_0–24 h_ for any of the interventions.

**Fig. 7:**
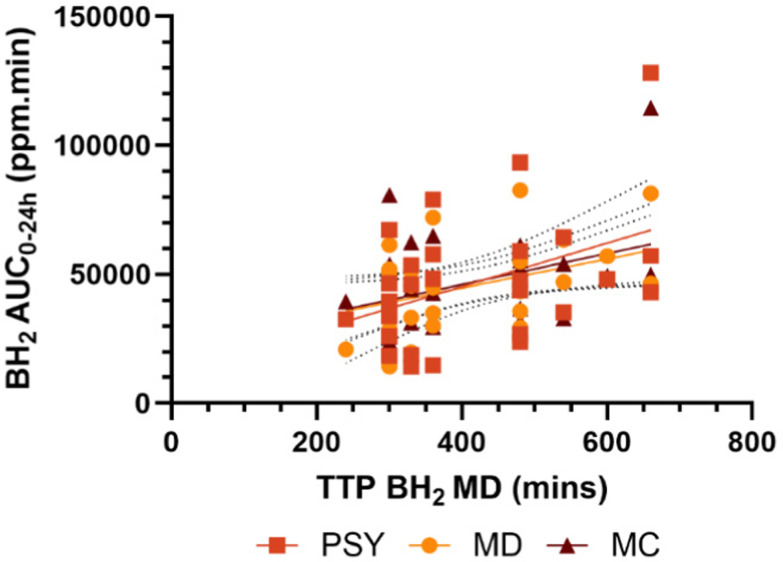
Correlation between TTP BH_2_ for MD against BH_2_ AUC_0–24 h_ of all three interventions.

Interestingly, the TTP BH_2_ for MD, reflecting the microbiota response without any gel-forming dietary fibre intervention, showed a wide range of values. There was a significant correlation of the TTP BH_2_ of MD with AUC_0–24 h_ for all 3 interventions; with MD (*r* = 0.42, *p* = 0.02), with PSY (*r* = 0.46, *p* = 0.03) and with MC (*r* = 0.48, *p* = 0.007) (Fig. 7).

## Discussion

Our approach was to emulate some of the physicochemical properties of PSY hydrogels using a more uniform material prepared from modified cellulose. We identified MC as the most promising for our goal; the thermoreversible gelation behaviour of MC is well documented.^22,34^ Attributes that can affect the sol–gel transition of MC include the average molecular weight and the degree of methylation^35–37^ as well as the presence of co-solutes.^38,39^

In all MC samples, the sol-to-gel transition temperatures were greater than the gel-to-sol transition temperatures. This indicates that, despite forming the cross-linked network at temperatures above body temperature (*i.e.* 37 °C), the gel-to-sol transition may not complete until samples are cooled below body temperature. This suggests that MC hydrogels may persist as a physically cross-linked hydrogel throughout the GI tract, although the extent of this MC gel structure retention is challenging to determine *in vivo*.

The *in vitro* model to assess the release of IN from dietary fibre hydrogels allowed screening of different MC formulations against our benchmark PSY hydrogel.^12^ Our optimal formulation was a combination of two different grades of MC, a low viscosity grade (A4 M, 4000 cP) and a high viscosity grade (MX, 40 000 cP), combined to a total of 4 w/w% in water, with comparable release rates to that of PSY. This combination showing a closer match to PSY in this test may be due to the combination of different hydrogel matrices from each grade of MC working in synergy to better mimic the heterogeneous polysaccharide matrix of PSY hydrogels.^40^

Considering the apparent complex viscoelastic behaviour of the MC and PSY hydrogels, while MC hydrogels had greater η* than PSY over the measured frequency range, the differences decreased with increasing angular frequency. Considering the viscoelastic contributions, namely *G*′ and *G*″, the distinct differences between the flow behaviours of the two hydrogels become more apparent. Specifically, the differences in *G*′ suggest a distinctly different extent of relaxation time between the two hydrogels, wherein their response to external stresses applied to MC hydrogels takes much longer to return to rest.

Having established that our MC hydrogel mimicked the release characteristics of PSY *in vitro*, we then tested its biological effects in a controlled human dietary intervention. We confirmed that PSY does slow orocaecal transit and reduce the BH_2_ in the first 6 hours (Fig. 4B), but does not significantly alter total fermentation assessed over 24 hours, compared to IN (Fig. 4C). By a small margin MC was not found to be non-inferior to PSY in reducing the early BH_2_ response to IN and its mode of action seems somewhat different. Our previous study showed that PSY's effect was due to both delayed arrival of the inulin bolus to the colon and further delayed fermentation in the first 9 hours in the colon.^17^ Despite MC likely increasing gastric chyme viscosity, unlike PSY it does not slow small bowel transit compared to MD (Fig. 5A). This may reflect its stimulation of small bowel secretion previously shown in perfusion experiments.^41^ The mechanism whereby MC stimulates small bowel secretion is unclear, but recent experiments in our laboratory using the same IN and MC intervention have confirmed this effect with an increase in small bowel water content along with increased water content in the ascending colon shown by MRI.^42^ The MC inhibitory effect on BH_2_ AUC_0–6 h_ appeared to depend strongly on the speed of small bowel transit, being much reduced in those with accelerated transit (Fig. 6). The wide variation in the effect suggests that the gel may break up in some subjects owing to their more vigorous motility which depends on many factors. As we have previously shown using agar gel beads of defined breaking strength,^43^ the propulsive forces which propel chyme through the narrow pylorus could disrupt the gel which could reduce inulin trapping. The superior effect of PSY may reflect the fact that owing to its “self-healing properties” after leaving the stomach it may reform the gel more effectively than MC.^40^ The two hydrogels show very similar frequency-dependent viscosity curves and exhibit viscoelastic solid behaviour based on storage (*G*′) and loss (*G*″) contributions (Fig. 3). PSY exhibited lower *G*′ values but greater *G*″ values than MC over the measured frequency range. This implies that PSY is more prone to deformation when under external forces applied to it, for example during peristalsis through the gastrointestinal tract. This deformation, while still existing as a viscoelastic solid, may be integral to the PSY hydrogel's ability to reform a continuous bolus throughout the GI tract.

Small intestinal transit depends on many luminal factors including viscosity, the balance of absorption/secretion and motility.^44^ The strong negative correlations we observed between OCTT and BH_2_ AUC_0–6 h_ for all 3 interventions suggest that early postprandial symptoms will depend on the rate of delivery of IN to the colonic bacteria (Fig. 6). Bacteria in the colon of a fasted subject will be deprived of carbohydrate and from the 24 hour profile we have previously reported after identical IN ingestion protocols^17^ and confirmed by the current study, BH_2_ production will be markedly reduced during fasting. The sudden switch to a substantial surplus could overwhelm the ability of bacteria to process reduced NADH other than by converting to hydrogen gas. Slowing delivery could allow utilisation of excess reducing equivalents to produce short chain fatty acids (SCFAs), which could lead to significant health benefits. The wide range in time to peak in the control group (MD) and its correlation with 0–24 h hydrogen production has not been reported before (Fig. 7). It is likely from subsequent MRI studies^42^ that the delayed peaks 6–8 hours after inulin ingestion reflect fermentation in the transverse rather than the ascending colon where the microbiota and their metabolic pathways may differ.

Limitations of this study include the lack of independent assessments of motility or transit since using the breath hydrogen response is confounded by the effect of the two fibres on fermentation. This will be addressed in subsequent publications using MRI,^14^ as well as using *in vivo* measures of colonic gas production and intracolonic transit using a telemetric capsule.^45^ Another constraint was our inability to track intracolonic gas production through direct sampling/measurement, which meant that we lacked overnight measurement of colonic fermentation. This will be addressed in part by the use of a telemetric capsule in an upcoming publication.^45^ Lastly, we acknowledge the difficulty is assessing what the shear forces are during passage through the pylorus and within the small intestine during postprandial mixing. Few authors have attempted to do this but de Loubens *et al.* concluded that shear was 0–0.4 s,^46^ so well within the ranges we tested *in vitro*.

Our results show that a mimic of PSY prepared using MC, despite both being physically cross-linked hydrogels showing similar flow behaviour and controlled release of IN, showed significantly different behaviour in the gut. We speculate that further modification of the cellulose preparation to reduce gel break down could be beneficial. This could be achieved by the addition of salts which can influence the gel-transition behaviour depending on their preferential solvation.^38^ It could also be achieved by combining methylcellulose with other cellulose derivatives, such as hydroxypropyl methylcellulose (HPMC) or carboxymethylcellulose (CMC).^47,48^ Alternatively, cellulose modified to feature multiple alkyl substitutions in block co-polymer arrangements has been shown to have distinctly different thermal gelation behaviour than comparable physical mixtures of alkyl cellulose derivatives.^49,50^ However, the impact of these food hydrocolloids on gastrointestinal health remains a hotly discussed area of research,^51,52^ with increasing concern over the long-term exposure to cellulose derivatives and increased risk of cardiovascular disease.^53^ Mechanistic understanding of the impact of these modified dietary fibres on intestinal barrier functions and colonic physiology is essential in understanding their risks and their benefits.^51,54^

## Conclusions

Despite the comparable physicochemical properties, our preparation of MC was not demonstrated to be non-inferior in reducing the initial fermentation of inulin compared to PSY in healthy volunteers. This may be partially explained by differences in intracolonic transit time and integrity of a continuous hydrogel network *in vivo*, as inferred by significant slowing of OCTT and time to peak BH_2_ for PSY compared to both MD and MC. Despite differences in initial fermentation of inulin, all three interventions showed comparable BH_2_AUC_0–24 h_, suggesting that inulin fermentation is delayed rather than prevented. The co-administration of a hydrogel dietary fibre with fermentable carbohydrates may be beneficial in improving their tolerance in patients living with IBS. For example, MC may be beneficial in patients with IBS with constipation (IBS-C) due to the acceleration of orocaecal transit, while PSY may be better suited to supporting patients with IBS with diarrhoea (IBS-D) due to slowing of orocaecal transit.

## Author contributions

Conceptualization: JR, AA, and RS. Data curation; JR, AA, TM, and DA. Formal analysis: JR, AA, CC, and RS. Funding acquisition: HH, FW, CH, PG, GY, MC, LM, and RS. Investigation: all authors. Methodology; all authors. Project administration: JR, AA, GY, MC, LM, and RS. Supervision: RS, MC, LM, and GY. Visualization; JR, AA, and RS. Writing – original draft: JR and RS. Writing – review & editing: all authors.

## Conflicts of interest

RS has received research grants from Sanofi & Nestle and is a consultant for Enterobiotix. MC is a consultant for Arena, Biocodex, PROMEDCS, Takeda, Nestle, RB, Mayoly. All other authors report no conflicts of interest.

## Disclaimer

The views expressed are those of the author(s) and not necessarily those of the NHS, the NIHR or the Department of Health and Social Care.

## Supplementary Material

FO-017-D5FO03532E-s001

## Data Availability

Supplementary information (SI): physicochemical characterisation data collected in this study. See DOI: https://doi.org/10.1039/d5fo03532e. Other original data will be made available from the corresponding author on reasonable request.
