# Supplementary material for: A randomised, placebo-controlled trial in healthy humans of modified cellulose or psyllium evaluating the role of gelation in altering colonic gas production during inulin co-administration
Source: Food Funct. 2026 Feb 4;17(4):1794–805. doi: 10.1039/d5fo03532e (PMC12869852; doi:10.1039/d5fo03532e)
Supplement: FO-017-D5FO03532E-s001 [file FO-017-D5FO03532E-s001.pdf]

Supplementary Information (SI) for Food & Function.
This journal is © The Royal Society of Chemistry 2026

A randomised, placebo-controlled trial in healthy humans of modified cellulose or psyllium evaluating the role of gelation in altering colonic gas production during inulin co-administration

Supplementary Information

Authors

Joshua E. S. J. Reid [1,2], Alaa T. Alhasani [2,3], Thomas MacCalman [1], Daniel Amor [1], Abdulsalam I. Aliyu [2], Amisha A. Modasia [4], Hannah Harris [4], Frederick J. Warren [4], Caroline Hoad [5], Penny A. Gowland [5], Gleb E. Yakubov [1,6], Colin Crooks [2], Maura Corsetti [2], Luca Marciani [2], Robin C. Spiller [2].

INSTITUTIONS (ALL):

1. Food Materials Research Group, School of Bioscience, University of Nottingham, Sutton Bonington, United Kingdom;

2. Nottingham NIHR Biomedical Research Centre and Nottingham Digestive Disease Centre, School of Medicine, University of Nottingham, Nottingham, United Kingdom;

3. Faculty of Health and Rehabilitation Sciences, Princess Nourah Bint Abdul Rahman University, Riyadh, Saudi Arabia;

4. Quadram Institute Bioscience, Norwich Research Park, Norwich, United Kingdom;

5. Sir Peter Mansfield Imaging Centre, School of Physics and Astronomy, University of Nottingham, Nottingham, United Kingdom;

6. Food Biopolymers Laboratory, School of Food Science and Nutrition, University of Leeds, Leeds, United Kingdom.

Preparation of dietary fibre solutions: physicochemical analysis

Inulin solution

For all formulations, a base solution containing inulin (4 w/w %) was prepared. Water (Reverse Osmosis purified) was freshly boiled and measured into a beaker (100.0 g) and placed on a stirrer plate. While the water was above 80°C, inulin (4.0 g) was gradually added to the beaker taking care to avoid formation of clumps. Once dissolved, the solution was either used straightaway or stored at 4°C in an airtight bottle and used within one week.

Psyllium solutions

Psyllium husks (2 – 4 w/w % final concentration in solution) were weighed out (accurate to 0.1 g). A quantity of the base inulin solution was measured into a beaker and heated to 50°C in a water bath. The psyllium husks were added completely to the solution, using a spatula to ensure complete incorporation. Solutions were left to cool to ambient temperature in air with occasional stirring to prevent phase separation (approximately 30 minutes). Once cooled, the solution was transferred to an airtight bottle, stored at 4°C and used within one week. Prior to analysis, samples were removed from storage and placed in a water bath set to an equilibrium temperature of 37°C.

Maltodextrin solutions

Maltodextrin (4 w/w % final concentration in solution) was weighed out (accurate to 0.1 g). A quantity of the base inulin solution was measured into a beaker and heated to 70°C with continuous stirring. Maltodextrin was gradually added to the solution taking care to avoid formation of clumps. Once fully dissolved, the solution was cooled to ambient temperature in air. Once cooled, solution was transferred to an airtight bottle, stored at 4°C and used within one week. Prior to analysis, samples were removed from storage and placed in a water bath set to an equilibrium temperature of 37°C.

Methylcellulose solutions

Quantities of methylcellulose (1 – 4 w/w % final concentration in solution) were weighed out (accurate to 0.1 g). A quantity of the base inulin solution was measured into a beaker and heated to a minimum of 70°C in a water bath. The methylcellulose was added completely to the solution, using a spatula to break up large clumps and create a homogenous dispersion. The beaker was secured into an ice bath with overhead stirring to continuously mix the solution as it cools. Once the solution had cooled to 10°C, it was transferred to an airtight bottle, stored at 4°C and used within one week.

To induce cross-linking, methylcellulose solutions were heated to between 60-70°C (dependent on methylcellulose composition) using a water bath. Care was taken to not exceed optimal gelation temperature which would result in syneresis. Once cross-linked, samples were transferred to a commercial blender for maceration into a fragmented gel mixture. The gel mixture was then transferred to a clean beaker and left to equilibrate to 37°C prior to analysis.

Physicochemical characterisation: methods and results

Differential scanning calorimetry

Thermal analysis of the methylcellulose preparations was performed using a micro differential scanning calorimeter (MicroDSC III, Setaram Instrumentation, France)[25]. Quantities (approximately 600 mg) of methylcellulose solutions were measured into Hastelloy cells. A reference cell contained reverse osmosis water of equivalent mass to the sample was used for each measurement. Samples were subjected to the following temperature program: 1) hold at 5°C, 5 minutes, 2) heat from 5°C to 95°C, 1°C/minute heating rate, 3) hold at 95°C, 5 minutes, 4) cool from 95°C to 5 °C, 1°C/minute cooling rate, repeating steps 1 – 4 for a total of two heat/cool cycles. The onset, peak and endpoint temperatures for both the sol-gel transition on heating and the gel-sol transition on cooling were determined using Calisto Processing software v1.43 (AKTS, Switzerland).

By comparing the gel transitions temperatures on both heating and cooling cycles using micro-DSC of MC, with and without IN, we sought to understand how IN may affect the formation and relaxation of the gel network of MC. Two different viscosity-grades of MC were assessed, as well as their mixture, to assess the association of flow properties with transition temperatures. A single concentration of MC was selected at 2% by weight to simplify comparison.

The thermal analysis results indicate that the lower viscosity grade MC (A4M) possessed higher transition temperatures than the higher viscosity grade MC (MX) (Table 1). For mixtures of the two MC varieties, the transition temperatures were between their pure components. With the addition of IN, all samples showed a slight change in the transition temperature range and peak: for the sol-to-gel transition, the temperatures decreased, while for the gel-to-sol transition the temperatures increased.

Hydrogel preparation	*T(gel, range) °C*	*T(gel, max) °C*	*T(sol, range) °C*	*T(sol, max) °C*
2% A4M	56.8 – 75.0	64.2	50.1 – 25.4	32.3
2% MX	49.7– 68.3	58.9	40.9 – 14.4	26.0
1% A4M + 1% MX	49.8 – 69.8	61.8	45.3 – 18.8	29.1
2% A4M + 2% IN	55.4 – 75.1	62.2	52.0 – 22.1	31.3
2% MX + 2% IN	47.6 – 62.6	57.2	47.0 – 12.4	27.0
1% A4M + 1% MX + 2% IN	52.9 – 68.2	60.1	44.6 – 17.4	28.3

Table S1: Transition temperatures (as range and max) for the sol-to-gel (T_{gel}) and for gel-to-sol (T_{sol}) transition.

In vitro model for Inulin release

An *in vitro* model was developed to assess the release of inulin from different formulations of MC against a positive control (PSY) and a placebo (MD). An orbital shaking incubator is set to 37°C with a beaker containing 500 mL of milliQ ultrapure water set inside and allowed to reach temperature. Test formulations (prepared as described above) were weighed into prepared dialysis tubing (BioDesign Dialysis tubing, 28.7 mm diameter, 14,000 MWCO, wetted in milliQ ultrapure water). The prepared dialysis tubes were sealed at each end using plastic clips to prevent spillage. For the formulations of methylcellulose and psyllium husk, a piping bag with a 10 mm diameter was used to dispense fibre gel into the dialysis tubing. Quantities varied between 50.0g and 55.0g of preparation for all measurements, determined from the difference of total mass and of the empty dialysis tubes and clips. The filled dialysis tube was transferred to the 500 mL beaker within the orbital shaking incubator. Samples were left shaking at a constant rate of 30 rpm. An aliquot (750 µL) of the dialysis water was removed at timed intervals of 0, 60, 120, 180, 240, 300 and 360 minutes. The experiment was repeated in triplicate to obtain biological replicates for each sample at each time point.

From these aliquots, the quantity of inulin present in the dialysis water was measured using spectrophotometry using an adapted method from Levine & Becker [26]. Samples from dialysis (600 µL) were combined with 1 w/w % vanillin solution (240 µL) in a 2 mL Eppendorf tube, lid replaced and shaken for 15 seconds. To the tube, 660 µL of concentrated sulfuric acid was added, lid replaced and shaken for a further 15 seconds, which resulted in exothermic reaction. Tubes were transferred to an ice bath and left to cool for 5 minutes. After this time approximately 1 mL of solution was transferred to a quartz cuvette using a Pasteur pipette. Samples were analysed using a Thermo Scientific Genesys 10S spectrophotometer at 520 nm. Quantitation was performed using inulin stock solutions (250.0 mg L^{-1}, serial diluted 1:2 down to 3.9 mg L^{-1}) and fitted to a standard curve.

Measured concentrations of inulin are converted into % of total inulin within mass of gel to standardise across replicates. The experiment was repeated four times in total for each analysed polysaccharide system, with standard deviations representing error bars (Figure 2). The data used to prepare this figure are summarised in the below table.

	Inulin Release (% of total inulin in gel)				
Sample	Time (min)	Repeat 1	Repeat 2	Repeat 3	Repeat 4
Blank	0	0	0	0	0
	60	5	6	4	4
	120	10	15	9	9
	180	19	20	14	13
	240	23	28	13	13
	300	27	30	21	21
	360	33	30	23	20
Maltodextrin, 4wt%	0	0	0	0	0
	60	3	3	6	6
	120	8	6	11	10
	180	10	8	15	14
	240	18	17	19	19
	300	22	19	22	23
	360	27	26	27	28
Psyllium, 4 wt%	0	0	0	0	0
	60	2	2	2	2
	120	4	3	3	3
	180	5	5	6	5
	240	5	5	8	7
	300	6	6	6	6
	360	8	9	8	8
Methylcellulose: A4M, 4 wt%	0	0	0	0	0
	60	3	2	1	2
	120	6	7	5	4
	180	9	9	8	6
	240	9	12	9	7
	300	12	13	11	9
	360	13	14	14	10
Methylcellulose: MX, 4 wt%	0	0	0	0	0
	60	2	2	2	2
	120	4	4	6	5
	180	6	5	6	6
	240	5	5	8	7
	300	9	9	8	8
	360	10	11	12	12
Methylcellulose: A4M, 2 wt% + MX, 2 wt%	0	0	0	0	0
	60	2	2	2	2
	120	3	3	3	3
	180	6	5	6	6
	240	5	5	5	5
	300	8	8	6	6
	360	7	8	8	8

Table S2: Replicate analysis of inulin release using *in vitro* dialysis model. Mean values and standard deviations represented on final figure.

Rheological characterisation

The viscoelastic flow properties of the dietary fibre hydrogel preparations were studied using an MCR 301 rheometer (Anton Paar GmbH, Graz, Austria). A cup (C-CC27/T200/SS, diameter 28.5 mm, effective depth 40.0 mm) and vane (ST22-4V-40, diameter 22.1 mm, effective depth 40.0 mm) geometry was utilised, with a horizontal gap of 6.4 mm and a vertical gap of 0.5 mm. A frequency sweep test was performed from 0.1 - 100 rad s^{-1} at constant strain of 1.00 % and held at 37.0°C using a Peltier temperature control system.

Angular Frequency [rad/s]	***G' [MC] (Pa)***			***G'' [MC] (Pa)***			***η* [MC] (mPa.s)***		
100.00	467.15	475.20	258.88	137.84	129.44	77.91	4.87	4.93	2.70
63.10	458.41	474.02	260.55	118.81	111.76	67.11	7.50	7.72	4.26
39.80	439.86	458.17	252.39	94.80	97.37	58.33	11.31	11.77	6.51
25.10	414.27	438.31	241.12	91.92	85.28	50.98	16.91	17.79	9.82
15.80	398.49	418.52	229.73	78.29	75.21	44.76	25.70	26.91	14.81
10.00	380.39	400.58	219.02	68.58	66.73	39.53	38.65	40.61	22.26
6.31	364.70	384.32	209.58	60.25	59.53	35.09	58.58	61.63	33.68
3.98	351.15	369.71	201.06	53.20	53.51	31.36	89.24	93.86	51.13
2.51	339.62	356.69	193.43	47.26	48.49	28.28	136.61	143.41	77.88
1.58	330.15	344.77	186.67	42.37	44.28	25.64	210.67	220.00	119.26
1.00	322.44	334.20	180.59	38.39	40.99	23.61	324.72	336.70	182.13
0.63	316.44	324.21	175.18	35.25	38.78	21.95	504.59	517.47	279.79
0.40	312.81	315.04	170.42	32.77	36.40	20.65	790.26	796.82	431.32
0.25	311.97	307.19	167.24	31.01	35.50	19.65	1249.03	1232.01	670.88
0.16	314.19	300.72	163.15	29.60	34.33	19.32	1997.35	1915.65	1039.81
0.10	319.03	294.51	160.24	28.85	34.51	19.09	3203.32	2965.25	1613.73

Table S3: Rheological characterisation of the methylcellulose (4wt%) and inulin (4wt%) hydrogel preparation utilised in the dietary intervention study. Mean values with standard deviations represented on figures.

Angular Frequency [rad/s]	***G' [PSY] (Pa)***			***G'' [PSY] (Pa)***			***η* [PSY] (mPa.s)***		
100.00	227.21	177.50	155.98	94.81	81.45	76.61	2.46	1.95	1.74
63.10	229.82	179.79	168.63	87.20	74.31	69.95	3.90	3.08	2.89
39.80	216.86	168.78	162.49	81.10	68.63	64.75	5.82	4.58	4.39
25.10	198.54	153.38	149.58	75.54	63.47	60.04	8.46	6.61	6.42
15.80	179.16	137.24	134.92	70.22	58.52	55.55	12.18	9.44	9.23
10.00	160.25	121.52	120.21	65.15	53.82	51.29	17.30	13.29	13.07
6.31	142.58	107.10	106.52	60.28	49.36	47.18	24.53	18.69	18.46
3.98	125.99	93.61	93.66	55.62	45.11	43.24	34.60	26.11	25.92
2.51	110.70	81.30	81.79	51.08	40.97	39.50	48.57	36.27	36.18
1.58	96.51	70.21	71.00	46.78	37.08	35.90	67.88	50.25	50.35
1.00	83.60	60.15	61.23	42.52	33.31	32.40	93.79	68.76	69.27
0.63	71.87	51.21	52.32	38.48	29.78	29.10	129.20	93.88	94.88
0.40	61.18	43.06	44.35	34.54	26.36	25.92	176.53	126.86	129.08
0.25	51.63	35.94	37.27	30.76	23.13	22.94	239.42	170.29	174.37
0.16	43.25	29.84	31.09	27.12	20.18	20.10	323.08	228.00	234.33
0.10	35.98	24.59	25.87	23.80	17.45	17.52	431.38	301.50	312.42

Table S4: Rheological characterisation of the psyllium (4wt%) and inulin (4wt%) hydrogel preparation utilised in the dietary intervention study. Mean values with standard deviations represented on figures.

Preparation of dietary fibre solutions: dietary intervention

Participants will be asked to consume or 375ml of intervention over a 20-minutes period. The test preparation will contain 15g inulin made up in 375 ml water with either;

1. 15 g psyllium husk
2. 15g methylcellulose
3. 15 g maltodextrin (placebo)

All test beverages will be prepared in advance of the study days using the Food Production Facility within the School of Biosciences, University of Nottingham. For all fibre drinks, an identical base drink containing Inulin (15g / 375 mL), stevia sweetener (0.1875 g / 375 mL) and strawberry flavouring (0.0375 g / 375 mL) is prepared by addition of all ingredients to freshly boiled water and with continuous mixing until all ingredients are dissolved.

For methylcellulose intervention: Methylcellulose (15g / 375 mL) is added to the base drink while hot (above 70°C) with continuous mixing to ensure it disperses within the base drink. The mixture is then allowed to cool to ambient temperature (20°C) where the solution thickens but remains pourable. The mixture is then poured into a polypropylene pouch so that the exact contents of the pouch is 375 mL (or equivalent mass of 420 g). The pouches are sealed using a thermal press. Pouches are then stored at 4°C and are to be consumed with one week. Any unused pouches (due to unforeseen circumstances) are to be disposed in general waste.

For Psyllium drink: Psyllium (15g / 375 mL) is added to the base drink while warm (between 40 - 50°C) with continuous mixing to ensure it disperses within the base drink. When the mixture has cooled to approximately 30°C, the solution will have thickened but remains pourable. The mixture is then poured into a polypropylene pouch so that the exact contents of the pouch is 375 mL (or equivalent mass of 420 g). The pouches are sealed using a thermal press. Pouches are then stored at 4°C and are to be consumed with one week. Any unused pouches (due to unforeseen circumstances) are to be disposed in general waste.

For maltodextrin drink: Maltodextrin (15g / 375 mL) is added to the base drink while hot (above 70°C) with continuous mixing to ensure it disperses within the base drink. The mixture is then allowed to cool to ambient temperature (20°C) where the solution thickens but remains pourable. The mixture is then poured into a polypropylene pouch so that the exact contents of the pouch is 375 mL (or equivalent mass of 420 g). The pouches are sealed using a thermal press. Pouches are then stored at 4°C and are to be consumed with one week. Any unused pouches (due to unforeseen circumstances) are to be disposed in general waste.

On the day of the study: For **Methylcellulose** drinks, the pouches need to be heated in a conventional microwave to set the gel (2:00 minutes upright, 20 seconds on side). After heating, the gels are allowed to attemperate to 37°C in a water bath for 15 minutes. For **Psyllium** and **Maltodextrin** drinks, the pouches are removed from the refrigerator and placed in a 37°C water bath for 15 minutes. The **Methylcellulose** and **Psyllium** fibre drinks will be divided into either **two or three portions of 125 mL** (depending on which study group the participant is in). The drink preparation will be done by an assistant who will not be involved in the data analysis to keep the study single blinded.

Intervention Study information

Recruitment & Screening

Healthy adult volunteers free from any preexisting gastrointestinal complaints were recruited after poster advertisement across the campuses of the University of Nottingham, UK. Participants attended an in-person screening session, where written consent, demographic information and any relevant medical information was obtained. In addition, participants consumed two muffins prepared with blue food dye and were asked to record the time when they pass stool of a distinctly different colour than normal to provide a measure of their usual whole gut transit time (WGTT). This assessment took place before participants took any intervention Participants were scheduled to three study days each separated by a minimum of 7 days for sufficient wash-out period. Instructions of dietary and lifestyle restrictions to be adhered to 24 hours before attending a study day until the morning after a study day (for a total of 48 hours) were provided. In total, 41 participants were randomised, with 30 participants completing all three study arms.

Randomisation and blinding

Eligible participants were randomised to their order of intervention to be consumed on a study day using Random.org. It was not possible to keep participants blinded to which intervention they consumed on a study day due to stark differences in appearance of each intervention. Single blinding of the intervention study was ensured by having a dedicated study team member prepare all interventions used in the study who was not involved in breath sample collection, data recording or data analysis. The blinding code was broken only after data analysis was completed.

Breath methane production

Of the 30 participants who completed the study, 8 participants produced methane on at least one breath test day with 2 participants producing methane on two breath test days and 3 participants on all three breath test days. No significant trends could be seen on the production of methane post-ingestion of any of the three interventions in this study.

Comment [rs]: @Joshua Reid (staff) lets put this in supplement just a distraction

Intervention study Consort Diagram

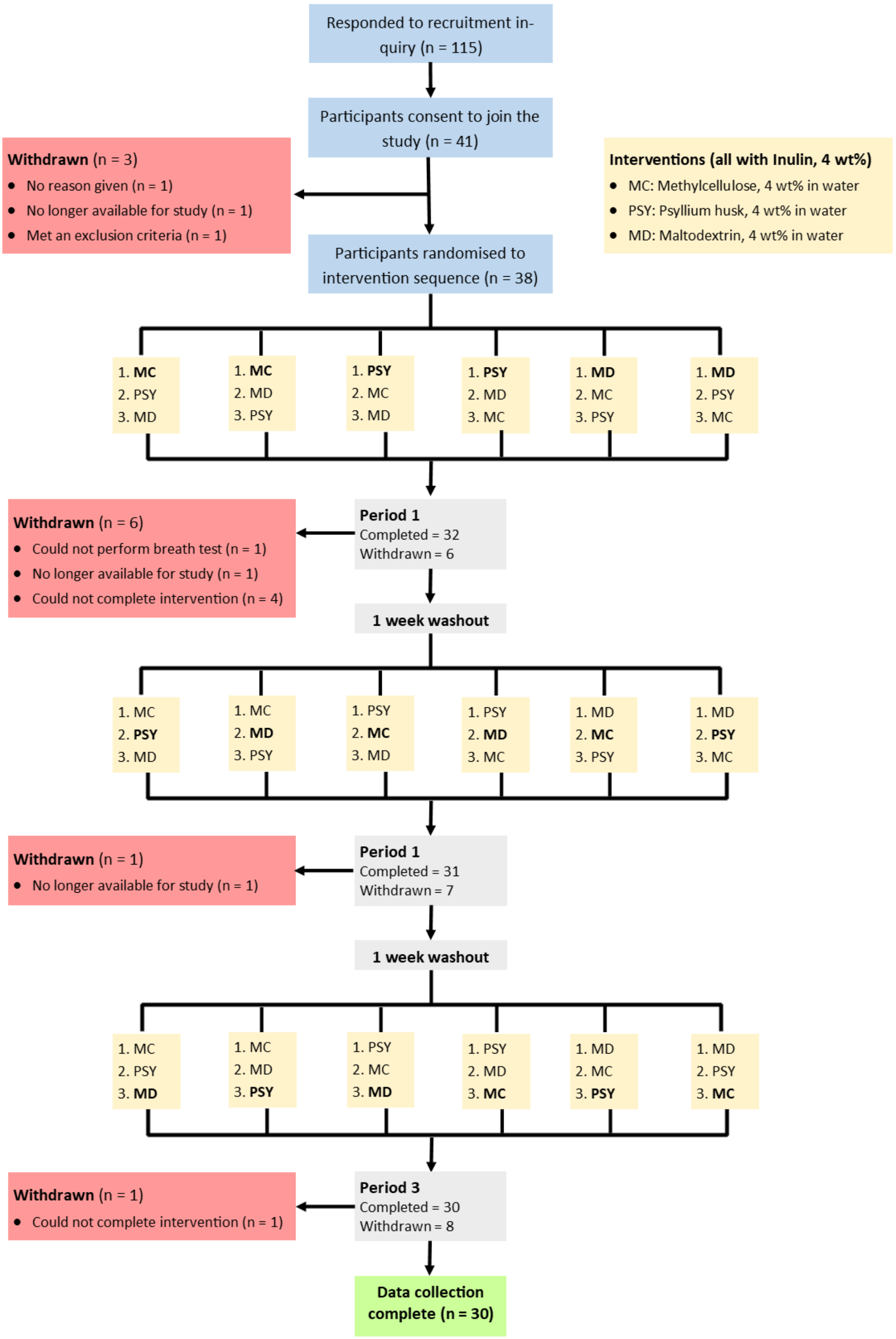

Figure S1: Consort diagram for recruitment and intervention schedule of participants in this study. In total, 41 volunteers attended screening sessions and provided written consent. After providing consent, 11 participants withdrew from the study: 5 participants could not consume the study intervention, 3 participants were no longer available for the study, 1 participant met an exclusion criterion (body mass index above 40.0), 1 participant could not perform the breath test, and 1 participant did not provide a reason. Thirty participants completed all three study arms.

Participant dietary and lifestyle guidance

Below is the excerpt from the guidance document supplied to participants in preparation and during a breath test study day.

Dietary restrictions the day before and during your study day:

- Avoided consuming certain food (e.g., beans, pulses, and lentils) before and during each study day. *Check the restricted items on the following page.*
- You may continue to drink water until you go to sleep.
- Please avoid excessive (>3/day) caffeine-containing drinks (tea, coffee, cola) on the day before your study day.
- Please avoid alcohol for 24 hours prior to your study days.
- Please avoid taking any energy drinks, dietary or sports supplement (e.g., multivitamins, iron supplements, protein shakes) for 24 hours prior to your study days.
- On the study day itself, please remain **nil by mouth** (do not eat or drink anything) until you reach the study centre. If you have essential medicines to take, then small sips of water are permitted to aid swallowing.

Lifestyle Restrictions the day before study day:

- Please avoid performing any strenuous exercise for 24 hours prior to your study day. This includes avoiding jogging or cycling briskly to the study centre on the day of your study.

NOTE:

- It is important that you stick to these restrictions before each study day as otherwise you will not be able to do the study that day.
- You should stick with these restrictions until you collect the last breath sample on the morning of the following day.

List of Restricted Food (and their alternatives) *

Food Group	Restricted items<br><br>(High FODMAPs**)	alternatives items<br><br>(Low FODMAPs**)
**Fruits**	Apples, apple juice, cherries, dried fruit, mango, nectarines, peaches, pears, plums, watermelon	Cantaloupe (melon), kiwi fruit (green), mandarin, orange, pineapple
**Vegetables**	Artichoke, asparagus, cauliflower, garlic, green peas, mushrooms, onion, sugarsnap peas/ Mange-touts	Aubergine/eggplant, beans (green), green capsicum (bell pepper), carrot, cucumber, lettuce, potato, zucchini/ courgette
**Dairy & alternatives**	Cow's milk, custard, evaporated milk, ice cream, soy milk (made from whole soybeans), sweetened condensed milk, yoghurt	Almond milk, brie/camembert cheese, feta cheese, hard cheeses, lactose-free milk, soy milk (made from soy protein)
**Protein sources**	Most legumes/pulses, some marinated meats/ poultry/ seafood, some processed meats	Eggs, firm tofu, plain cooked meats /poultry/ seafood
**Breads & cereals**	Wheat/rye/barley-based breads, breakfast cereals, biscuits, and snack products	Corn flakes, oats, quinoa flakes, quinoa/rice/corn pasta, rice cakes (plain), sourdough spelt bread, wheat/rye/barley free breads
**Sugars, sweeteners & confectionery**	High fructose corn syrup, honey, sugar free confectionery	Dark chocolate, maple syrup, rice malt syrup, table sugar
**Nuts & seeds**	Cashews, pistachios	Macadamias, peanuts, pumpkin seeds/pepitas, walnuts

***Reference**: https://www.monashfodmap.com/about-fodmap-and-ibs/high-and-low-fodmap-foods/

** FODMAPs is refers to Fermentable Oligosaccharides, Disaccharides, Monosaccharides, and Polyols. FODMAPs are a class of carbohydrates that are neither digested nor absorbed in the small intestine, rather fermented in the colon by microbiota. FODMAPs are known to exacerbate GI symptoms (bloating and abdominal pain) in most IBS patients due to their functional characteristics.

*** Download (**FODMAP A to Z**) application from your phone store to enable you to know the FODMAPs category (low or high) of different food items.

- Apple users: https://apps.apple.com/us/app/low-fodmap-diet-a-to-z/id1356683228
- Google play store: https://play.google.com/store/apps/details?id=uk.co.temeraire1798.fodmapaz&hl=en&gl=US

Statistical analysis of breath hydrogen time curve

Two-way RM ANOVA	Matching: Stacked				
Assume sphericity?	No				
Alpha	0.05				
**Source of Variation**	**% of total variation**	**P value**	**P value summary**	**Significant?**	**Geisser-Greenhouse's epsilon**
Time x Fibre treatment	2.335	0.0234	*	Yes	0.2023
Time	32.27	<0.0001	****	Yes	0.2023
Fibre treatment	0.4749	0.3682	ns	No	
Subject	20.44	<0.0001	****	Yes	
**ANOVA table**	**SS**	**DF**	**MS**	**F (DFn, DFd)**	**P value**
Time x Fibre treatment	31622	38	832.2	F (7.689, 334.5) = 2.283	P=0.0234
Time	437014	19	23001	F (3.845, 334.5) = 63.11	P<0.0001
Fibre treatment	6432	2	3216	F (2, 87) = 1.011	P=0.3682
Subject	276782	87	3181	F (87, 1653) = 8.729	P<0.0001
Residual	602476	1653	364.5		

Data summary	
Number of columns (Fibre treatment)	3
Number of rows (Time)	20
Number of subjects (Subject)	90
Number of missing values	0
